# Supplementary material for: An ancestral apical brain region contributes to the central complex under the control of foxQ2 in the beetle Tribolium
Source: eLife. 2019 Oct 18;8:e49065. doi: 10.7554/eLife.49065 (PMC6837843; doi:10.7554/eLife.49065)
Supplement: Supplementary file 1. [file elife-49065-supp1.docx]

**Table 1. Number of Tc-FoxQ2^+^ neural precursors (NPCs) per half-side during embryogenesis.**

| **Stage/Individual** | | **#1** | | **#2** | | **#3** | | **#4** | | **#5** | | **#6** | | **Mean** |
| --- | --- | --- | --- | --- | --- | --- | --- | --- | --- | --- | --- | --- | --- | --- |
| **NS8** | 15 | | 16 | | 15 | | 12 | | 17 | | 16 | | 15 | |
| **NS11** | 10 | | 9 | | 9 | | 11 | | 12 | | 10 | | 10 | |
| **NS14** | 6 | | 5 | | 6 | | 5 | | 7 | | 5 | | 6 | |

**Table 2. gRNAs target sequences and oligonucleotides used for generating gRNAs.** The PAM sequence is marked in red. The orange sequence represents the complementary overhangs to the vector generated by *BsaI* digestion.

| **Name of gRNA** | **Genomic target sequence** | **Sense oligo** | **Antisense oligo** |
| --- | --- | --- | --- |
| **gRNA1** | GGGCGTTACTGTCACCCTCCAGG | TTCGGGCGTTACTGTCACCCTCC | AAACGGAGGGTGACAGTAACGCC |
| **gRNA2** | GTGGCGGGGCGGAGCCAACGCGG | TTCGTGGCGGGGCGGAGCCAACG | AAACCGTTGGCTCCGCCCCGCCA |
| **gRNA-eb** | GAACCGGGCAGCCCGCCTCCTGG | TTCGAACCGGGCAGCCCGCCTCC | AAACGGAGGCGGGCTGCCCGGTT |

**Table 3. Plasmids used as PCR templates and molecular cloning.**

| **Plasmid and internal number** | **Description** |
| --- | --- |
| pJET1.2 | backbone for knock-in construct cloning |
| # 82 pBac[3xP3-gTc'v; Tc'ems-overex] | used to amplify 3xP3, *Tc-vw* |
| # 169 pSLfa[Tc-hsp_p-ECFP-SV40] | used to amplify SV40 |
| # 133 pSLfa[Tc’hsp5’-Cre recomb-3’UTR]fa | used to amplify Cre |
| # 65 pBac[3xP3-gTc'v; Tc'hsp-Promotor RC] | used to amplify bhsp |

**Table 4. Number of the injected embryos, the hatched larvae and developed adult beetles as well as the efficiency of germ line transmission.**

| **Target region** | **Injected embryos** | | **Hatched larvae** | | **Hatching rate[%]** | | **Developed adults** | | | **Survival rate [%]** | | **Efficiency [%]** |
| --- | --- | --- | --- | --- | --- | --- | --- | --- | --- | --- | --- | --- |
| **Upstream** | | 1496 | | 479 | | 31.99 | | 234 | 15.64 | | 6/234=2.6 | |

**Table 5. Number of cells in the *anterior-median-foxQ2-cluster*.**

| **Stage/Ind.** | | **#1** | **#2** | **#3** | **#4** | **#5** | **Mean** |
| --- | --- | --- | --- | --- | --- | --- | --- |
| **NS13** | **Total cells** | 89 | 93 | 84 | 85 | 96 | 89 |
|  | **NPCs** | 12 | 11 | 13 | 14 | 13 | 12 |
|  | **Weak cells** | 13 | 10 | 12 | 15 | 10 | 12 |
| **NS14** | **Total cells** | 154 | 142 | 150 | 149 | 158 | 150 |
|  | **NPCs** | 11 | 15 | 14 | 13 | 16 | 14 |
| **NS15** | **Total cells** | 219 | 210 | 230 | 238 | 225 | 224 |

**Table 6. Number of cells in the *anterior-lateral-foxQ2-lineage*.**

| **Stage/Ind.** | | **#1** | | **#2** | | **#3** | **#4** | | **Mean** | |
| --- | --- | --- | --- | --- | --- | --- | --- | --- | --- | --- |
| **NS14** | 8 | | 6 | | 7 | | | 6 | | 7 |
| **NS15** | 15 | | 17 | | 18 | | | 20 | | 18 |

**Table 7. Number of cells in respective groups of** ***Ten-a-green* line.**

| **Location/Ind.** | | **#1** | **#2** | **#3** | **#4** | **Mean** |
| --- | --- | --- | --- | --- | --- | --- |
| **Anterior group** | **WT** | 38 | 40 | 41 | 36 | 39 |
|  | **RNAi** | 22 | 28 | 17 | 16 | 20 |
| **Posterior-lateral group** | **WT** | 36 | 31 | 27 | 34 | 32 |
|  | **RNAi** | 7 | 16 | 22 | 17 | 16 |
| **Posterior-median group** | **WT** | 30 | 28 | 27 | 24 | 27 |
|  | **RNAi** | 8 | 17 | 15 | 18 | 15 |
| **Total number** | **WT** | 104 | 99 | 95 | 94 | 98 |
|  | **RNAi** | 37 | 61 | 54 | 51 | 51 |

**Table 8. Number of cells of *Tc-rx* line.**

| **Location/Ind.** | | **#1** | **#2** | **#3** | **#4** | **#5** | **#6** | **Mean** |
| --- | --- | --- | --- | --- | --- | --- | --- | --- |
| **Anterior-median group** | **WT** | 35 | 42 | 43 | 36 | 41 | 44 | 40 |
|  | **RNAi** | 11 | 9 | 7 | 11 | 12 | 6 | 9 |

**Table 9. Number of cells of *foxQ2-5’-line*.**

| **Anterior-median cluster and lateral lineage** | | **#1** | **#2** | **#3** | **#4** | **Mean** |
| --- | --- | --- | --- | --- | --- | --- |
| **NS13** | **WT** | 89 | 93 | 96 | 85 | 89 |
|  | **RNAi** | 45 | 50 | 28 | 43 | 42 |
| **NS15** | **WT** | 227 | 250 | 253 | 243 | 243 |
|  | **RNAi** | 104 | 95 | 120 | 117 | 109 |

**Table 10. Primer sequences and purposes used for cloning.**

| Primer | Sequence ( 5’-3’) | Purpose |
| --- | --- | --- |
| BH_C_ter_fwd | CCAGGTCTCATGGTTCCACGTCCGTTTATCACAC | Cloning of *Tc-foxQ2* C-terminal fragment with *BsaI* |
| BH_C_ter_rev | GGGGGTCTCCTCGAGTTAAGAGTCTGTGGTGTCGGTGGC |  |
| RT1_EGFP_bhsp_rev | TGCTCACCATGTTTGACTTTGAATTCACTAGTAAATAATTCACTCAACTTTGTTAAAG | Cloning of bhsp, EGFP, 2A and Cre |
| RT2_bhsp_EGFP_fwd | AGTGAATTCAAAGTCAAACATGGTGAGCAAGGGCG |  |
| RT3_2A_EGFP_rev | GTCTCCTGCTTGCTTTAACAGAGAGAAGTTCGTGGCTCCGGATCCCTTGTACAGCTCGTCCATGCC |  |
| RT4_2A_Cre_fwd | GCCACGAACTTCTCTCTGTTAAAGCAAGCAGGAGACGTGGAAGAAAACCCCGGTCCTATGTCCAATTTACTGACCGTACACCAA |  |
| RT5_3xP3_fwd | GATTCTAGACATTATTCATTAGAGACTAATTCAATTAGAGCTAATTCAATTAGGATCC | Cloning of 3xP3, vermillion and Sv40 |
| RT6_Sv40_Cre_rev | AATGGAAACAATTAAGATGAGTTTGGACAAACCACA |  |
| RT7_Sv40_Cre_fwd | TTGTCCAAACTCATCTTAATTGTTTCCATTCGACACGT |  |
| RT8_Sv40_vw_rev | GTATGGCTGATTATGACTAATCGCCATCTTCCAGCA |  |
| RT9_Sv40_vw_fwd | GGAAGATGGCGATTAGTCATAATCAGCCATACCACA |  |
| RT10_ebony_fwd | GTCGGGCCCGAACCGGGCAGCCCGCCTCCTGGCGTTTCATATATAAGCGCGGTCTCG | Cloning of ebony site with *ApaI* |
| BH_AG _fwd | GCGCTGGCATTTTTAAATCACG | Testing the  insertion site |
| BH_AG_rev | ATACTGTAGAGCTGGAGCC |  |
| BH_EGFP_rev | TGGTGCAGATGAACTTCAG |  |
| rx_fwd | ACGGATCCGGGATCAAGCGTAAATGGGACGTCCCATACAATA | Cloning of *Tc-rx* 5’up regulatory region with *BamHI*, *NheI* |
| rx_rev | ATCTACGCTAGCCTTCACAACGGTCCGATTCTATCGC |  |
| DsRed_fwd | ATGGCCTCCTCCGAGGA | Cloning of DsRedExpress |
| DsRed_rev | CTACAGGAACAGGTGGTGG |  |
