# Supplementary material for: An ancestral apical brain region contributes to the central complex under the control of foxQ2 in the beetle Tribolium
Source: eLife. 2019 Oct 18;8:e49065. doi: 10.7554/eLife.49065 (PMC6837843; doi:10.7554/eLife.49065)
Supplement: Transparent reporting form [file elife-49065-transrepform.pdf]

1st Floor
24 Hills Road
Cambridge CB2 1JP, UK

P 01223 855340
W elifesciences.org
T @elife

# *eLife's* transparent reporting form

We encourage authors to provide detailed information *within their submission* to facilitate the interpretation and replication of experiments. Authors can upload supporting documentation to indicate the use of appropriate reporting guidelines for health-related research (see EQUATOR Network), life science research (see the BioSharing Information Resource), or the ARRIVE guidelines for reporting work involving animal research. Where applicable, authors should refer to any relevant reporting standards documents in this form.

If you have any questions, please consult our Journal Policies and/or contact us: editorial@elifesciences.org.

**Sample-size estimation**

- You should state whether an appropriate sample size was computed when the study was being designed
- You should state the statistical method of sample size computation and any required assumptions
- If no explicit power analysis was used, you should describe how you decided what sample (replicate) size (number) to use

Please outline where this information can be found within the submission (e.g., sections or figure legends), or explain why this information doesn't apply to your submission:

Our developmental study did not include experiments where we considered statistical sample size estimation reasonable. The sample size of the knock-down embryos was balanced with the effort to stain, prepare and analyze the data, respectively (e.g. by LSM imaging and manual quantification of marked cells). For embryonic stainings we usually stained >50 embryos stemming from a collection of more than 100 females in parallel and used 5-6 representative individuals per stage for careful quantification. For brains, we usually stained >5 in parallel and analyzed 4-6 by LSM imaging.

The number of animals analyzed is given in the results text when referring to the respective Figure. E.g. "(n=6; Fig. 1A; Table S1)"

**Replicates**

- You should report how often each experiment was performed
- You should include a definition of biological versus technical replication
- The data obtained should be provided and sufficient information should be provided to indicate the number of independent biological and/or technical replicates
- If you encountered any outliers, you should describe how these were handled
- Criteria for exclusion/inclusion of data should be clearly stated
- High-throughput sequence data should be uploaded before submission, with a private link for reviewers provided (these are available from both GEO and ArrayExpress)

Please outline where this information can be found within the submission (e.g., sections or figure legends), or explain why this information doesn't apply to your submission:

We performed most experiments once using a large number of treated animals. In parental RNAi experiments, we injected 250-300 pupae and collected from them >50 embryos for staining. In larval and adult experiments, we stained >5 brains. 4-6 brains were analyzed by LSM imaging.
For *in vivo imaging*, we performed each setup twice (40X magnification from one perspective and 10X magnification from several perspectives). All films document the same process (development of EGFP positive cells) adding to a total of four independent repetitions.
We considered only phenotypes that we saw in several independent specimen.

We mention this in the materials and methods.

**Statistical reporting**

- Statistical analysis methods should be described and justified
- Raw data should be presented in figures whenever informative to do so (typically when N per group is less than 10)
- For each experiment, you should identify the statistical tests used, exact values of N, definitions of center, methods of multiple test correction, and dispersion and precision measures (e.g., mean, median, SD, SEM, confidence intervals; and, for the major substantive results, a measure of effect size (e.g., Pearson's r, Cohen's d)
- Report exact p-values wherever possible alongside the summary statistics and 95% confidence intervals. These should be reported for all key questions and not only when the p-value is less than 0.05.

Please outline where this information can be found within the submission (e.g., sections or figure legends), or explain why this information doesn't apply to your submission:

We noted the number of animals that was checked in the results section when referring to the respective figure and we give the exact numbers in a supplementary table 1. The number of animals was too low for statistical testing.
For example: "(n=6; Fig. 1A; Table S1)"

(For large datasets, or papers with a very large number of statistical tests, you may upload a single table file with tests, Ns, etc., with reference to sections in the manuscript.)

**Group allocation**

- Indicate how samples were allocated into experimental groups (in the case of clinical studies, please specify allocation to treatment method); if randomization was used, please also state if restricted randomization was applied
- Indicate if masking was used during group allocation, data collection and/or data analysis

Please outline where this information can be found within the submission (e.g., sections or figure legends), or explain why this information doesn't apply to your submission:

There are essentially two groups of treatment: Wildtype animals (not treated by RNAi) and experimental animals (treated with RNAi). This information is given in the results text.

**Additional data files ("source data")**

- We encourage you to upload relevant additional data files, such as numerical data that are represented as a graph in a figure, or as a summary table
- Where provided, these should be in the most useful format, and they can be uploaded as "Source data" files linked to a main figure or table
- Include model definition files including the full list of parameters used
- Include code used for data analysis (e.g., R, MatLab)
- Avoid stating that data files are "available upon request"

Please indicate the figures or tables for which source data files have been provided:

We have uploaded the original LSM stacks and processed versions thereof (.avi format) of most figures and supplementary figures to figshare. This is indicated in the text and in materials and methods. We have integrated the construct for enhancer trapping via genome editing and its sequence to AddGene. We have uploaded the original data of the in vivo imaging and the respective metadata to Zenodo.

The details are given in our section "data availability":
"All LSM stacks can be downloaded from the figshare repository (https://figshare.com/account/home#/projects/62939). The construct used for generating the enhancer trap is available from AddGene (#124068). The in vivo imaging data is accessible at Zenodo (10.5281/zenodo.2645645 Dataset DS0001 / "left part" of Figure 6 and Supplementary Movie X; 10.5281/zenodo.2645657 Dataset DS0002; 10.5281/zenodo.2645665 Dataset DS0003 579 / "right part" of Figure 6 and Supplementary Movie X+1)"

eLife Sciences Publications, Ltd is a limited liability non-profit non-stock corporation incorporated in the State of Delaware, USA, with company number 5030732, and is registered in the UK with company number FC030576 and branch number BR015634 at the address 1st Floor, 24 Hills Road, Cambridge CB2 1JP | August 2014
